# A perspective on developing foundation models for analyzing spatial transcriptomic data

**DOI:** 10.1002/qub2.70010

**Published:** 2025-06-13

**Authors:** Tianyu Liu, Minsheng Hao, Xinhao Liu, Hongyu Zhao

**Affiliations:** ^1^ Interdepartmental Program of Computational Biology and Bioinformatics Yale University New Haven Connecticut USA; ^2^ Department of Biostatistics Yale University New Haven Connecticut USA; ^3^ Research and Early Development Genentech South San Francisco California USA; ^4^ Department of Computer Science Princeton University Princeton New Jersey USA; ^5^ Present address: Department of Biomedical Informatics Harvard University Boston Massachusetts USA.

**Keywords:** artificial intelligence, foundation models, perspective, spatial transcriptomics data

## Abstract

Do we need a foundation model (FM) for spatial transcriptomic analysis? To answer this question, we prepared this perspective as a primer. We first review the current progress of developing FMs for modeling spatial transcriptomic data and then discuss possible tasks that can be addressed by FMs. Finally, we explore future directions of developing such models for understanding spatial transcriptomics by describing both opportunities and challenges. In particular, we expect that a successful FM should boost research productivity, increase novel biological discoveries, and provide user‐friendly access.

## 1 INTRODUCTION

Foundation models (FMs) [1], within the scope of deep learning, are defined as models pretrained with large‐scale corpus data and can be utilized to address various problems (known as downstream applications) [2, 3, 4]. Such models have achieved significant success in accelerating scientific discoveries, especially in natural language processing (NLP) handled by large language models (LLMs) [2] as well as multimodal data processing handled by large multimodal models (LMMs) [3]. However, in the area of genomics, especially in spatial transcriptomic data analysis, we have yet to find FMs that are capable of generating novel and validated discoveries.

The success of FMs in modeling natural language and multimodal data largely benefits from the advancement of a model architecture, known as the transformer [5], which is capable of capturing the information in sequence data efficiently. Additionally, training FMs in these two areas is also supported by carefully selecting high‐quality training data [6, 7]. Therefore, we should expect to transfer the success of FMs in the NLP area to biomedical analysis if the biomedical data share a similar structure and quality with natural language data. However, spatial transcriptomics (ST) contains two sets of information: the gene expression information as well as the coordinates of spots, which do not have an explicit sequence‐like data structure and are also noisy [8, 9]. Such problems also exist in building FMs for analyzing single‐cell transcriptomics data, which does not have the spatial information for each cell. For single‐cell data, researchers have developed single‐cell FMs by pretraining the base model with large‐scale single‐cell transcriptomics data [10, 11, 12]. However, it has been shown [13, 14, 15, 16] that the performance of single‐cell FMs is not proportional to their consumed resources and may not be defined as FMs.

In this perspective, we summarize the current progress on developing FMs for analyzing ST data. We propose problems that align with the requirement of developing FMs and discuss their future developments.

## 2 DEFINING A FM

**FIGURE 1:**
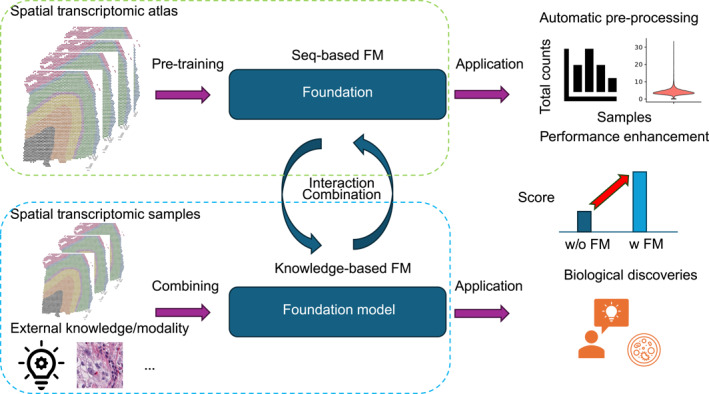
Illustration of two paradigms for building FMs for spatial transcriptomic analysis. The seq‐based FM is highlighted in the green block, and the knowledge‐based FM is highlighted in the blue block. We also include examples of preprocessing steps and contributions of these FMs in the right panel. FMs, foundation models.

FMs for ST analysis can be classified into two different types: one type is driven by sequencing data (seq‐based FMs), and another type is driven by prior biological knowledge (knowledge‐based FMs). These two types of FMs have shared tasks and training frameworks but differ in ideas, resource consumption, and training objectives. The two different paradigms are summarized in Figure 1.

Although we can use scRNA‐seq‐based FMs to address the research questions in ST data, there are significant differences between these two types of FMs. First, scRNA‐seq data and ST data have different resolutions and distributions [17]. The noise of ST data can be caused by factors such as sequencing errors, mismatched niche annotation, unmeasured important genes, wrong cell segmentation, and other factors. Therefore, the preprocessing and data‐cleaning approaches used for scRNA‐seq data cannot be directly transferred to ST data. Improving sequencing technology, cell segmentation method, and imputation approach might reduce the noise level of ST data. Second, ST data have more information than scRNA‐seq, such as spatial location, and it is necessary to consider spatial context at least for certain cell types [18] to build a successful FM. Third, ST data have a higher cost than scRNA‐seq data, and thus building an FM for analyzing ST data requires a higher standard for constructing the training corpus [19]. Therefore, we need to consider a new paradigm for building FMs.

For seq‐based FMs, researchers pretrained the model with large‐scale ST data and utilized the pretrained model to handle various downstream applications with either fine‐tuning or zero‐shot learning. To train a seq‐based FM, researchers utilize sequencing data with location information, and the training objective is usually self‐supervised learning [20]. As an example, NicheCompass [21] is an autoencoder pretrained with large‐scale ST data. During the training process, the known cell–cell interactions are transformed into covariate embeddings to guide the model’s learning. Here, a “niche” is defined as a group of spots co‐localized by following certain geometric rules. NicheCompass is capable of various downstream applications, including spatial atlas building, niche identification, niche classification, dataset‐specific cell–cell communication inference, and niche query. Similarly, Novae [22] models spatial data with a graph‐aware encoder, but its training loss relies on contrastive learning. Another FM, Nicheformer [23], models spatial coordinates as a different modality separate from gene expression and defines the spatial modality as a token in the pretraining process of their Nicheformer transformer blocks with diverse ST data. Through fine‐tuning, Nicheformer is capable of spatial label identification, spatial composition deconvolution, and predicting changes in cellular neighborhood density. Moreover, STFormer [24] models niche‐level information with gene expression information jointly and proposes the recovery of gene–gene interaction as a new creative pretraining objective. SpaFormer [25] proposes a new transformer‐based autoencoder framework for imputing ST data. Lastly, considering the similarity between single‐cell transcriptomics and ST, CellPLM [26] also treats cells as tokens and encodes spatial coordinates with positional encoding in their pretraining stage. It utilizes ST as data augmentation of single‐cell transcriptomics to enhance its function in batch effect correction, cell‐type annotation, perturbation prediction, and spatial imputation. Overall, seq‐based FMs leverage the advantages of large‐scale data and advanced model architecture for model development.

For knowledge‐based FMs, researchers incorporated LLMs or LMMs for processing ST data, as LLMs could be pretrained with text‐based biological knowledge [27], and LMMs could be pretrained with biomedical images [28]. Developing knowledge‐based FMs requires data from different modalities but might not necessarily pretrain a new base model or fine‐tune a model for downstream applications. As an example, QuST‐LLM [29] combines QuST as a pioneering tool for processing images and LLM as an advanced tool for processing text‐based gene pathway information. Such a design can help researchers uncover spatial insights at the niche level. Moreover, Geneverse [30] considers fine‐tuning existing LMMs with expression patterns of genes in ST data to identify marker genes of different cell types. Overall, knowledge‐based FMs leverage the advantages of general FMs designed for other purpose and transfer the knowledge to analyze ST.

The interaction between seq‐based FM and knowledge‐based FM is also emerging, as knowledge can be interpreted as a new modality in the model training stage and thus the capacity of these FMs is likely to be enhanced. For example, spEMO [31] leverages pathology FMs to encode image embeddings with transcriptomic embedding jointly, and thus we can also learn the contextual information from other modalities to analyze sequencing data and perform survival prediction and disease‐state classification. scGPT‐spatial [32] is developed based on the pretrained scGPT with single‐cell transcriptomics data, which has been shown to have promising results in data integration, deconvolution, and gene expression imputation. Therefore, the combination of these two types of FMs might play an important role in building the technical routes for modeling atlas‐level transcriptomic data.

## 3 DISCUSSING THE NEED OF FM

Given the cost of training and the expectation of the capabilities of FMs, we believe that the capacity of an FM should be stronger than handling simple tasks [13, 33, 34], such as spot‐level annotation or clustering on data with simple structure or low annotation resolution [35]. Instead, it should focus on addressing the major limitations of current methods, for example, identifying noncontinuous spatial domains and accelerating novel biological discovery. By incorporating the general perspective of developing an FM, a suitable FM should be utilized for handling the following tasks: it either can help perform automatic selection of the optimal preprocessing steps or provide interpretation for driving novel biology discoveries.

The data preprocessing steps have been extensively studied by researchers [36]. Because of the noise level of ST data, the conclusions of analyzing such data are always strongly affected by the choices of quality control, data normalization, integration, clustering, and annotation [37, 38]. Currently, we are guided by empirical approaches for the preprocessing steps, and the choices made by different researchers are subjective. Therefore, a good FM should unify the preprocessing stage of different ST data and select the best option for each step, which can be performed by data integration or harmonized cell‐type (or niche‐type) annotation. Such a design can not only guarantee the best utilization of known datasets but also ensure reproducibility.

Using prior knowledge from FMs to enhance the performances of domain‐specific models is also important. In the analyses of single‐cell transcriptomics, methods such as GenePT [39] and scELMo [40] have demonstrated that using text embeddings from LLMs can improve the performance of current models in several downstream tasks, including cell‐type annotation, drug response prediction, and perturbation prediction. Furthermore, scFoundation [11] also showed that incorporating cell embeddings or gene embeddings from pretrained single‐cell FMs can help with downstream tasks. However, we have not seen much exploration of the applications related to ST, such as spatial developmental biology [41] or spatial tumor microenvironment [42]. For spatial data, we believe that there are several opportunities for FMs to improve the performance. For instance, in the cell‐type annotation task, FM could be fine‐tuned to improve the classification accuracy, usually measured by the F1 score. In the spatial niche clustering task, FM could generate embeddings for niches and improve the adjusted mutual information (AMI) score or average silhouette width (ASW) [43]. In the spatial gene imputation task, FM could enhance the signal‐to‐noise ratio. Common evaluation metrics are correlation and cosine similarity between predicted and ground truth expression. Spatial deconvolution is another common downstream task in ST, where FM is able to improve the accuracy score, including Pearson correlation, structural similarity index (SSIM), root mean square error (RMSE), and Jensen–Shannon divergence (JS) [44]. Meanwhile, how to incorporate multimodal information from FMs to boost current design presents a promising area for future research.

Using AI models to accelerate the process of biological discovery is another possible major contribution. Biological experiments consume more resources than in silico experiments. This is a direct motivation for applying AI models to help with experiment validation [45]. For example, ChemCrow [46] introduced a new AI agent for augmenting the LLM performance in chemistry to help discover new molecules. It would be interesting to have a similar AI agent for processing ST data. FMs can also be used to identify novel cell types or predict perturbation effects. UCE [47] demonstrated an example of identifying new cell types to advocate the necessity of developing single‐cell FMs. Specifically, there has been little research to explore perturbation analysis based on ST data or to fully explain the contributions of spatially induced patterns. Therefore, it is important to incorporate the design for novel biological discovery, as well as the comparison between the cost of human resources and model training, during the development of FMs for analyzing ST data.

Finally, we note that these two types of FMs for ST data analysis also have their own limitations. For example, sequencing‐based FMs typically require data from diverse resources for training to learn a better contextual representation, and thus data collection as well as storage becomes challenging. Moreover, knowledge‐based models might require developers to collect more knowledge‐enriched datasets or multimodal data, and biological discovery might be constrained by the prior information. Evaluating the performance of these FMs in real downstream applications is also challenging, as we expect to cover as many cases as possible in the validation step.

## 4 EXPLORING THE FUTURE OF FM

In this perspective, we defined the concept of an FM for ST data analysis, followed by possible tasks that need the capacity of FMs to address. However, regarding possible applications, many challenges are presented. First, we should pay more attention to collect high‐quality and diverse sequencing‐based data and knowledge‐based data, which will be the important basis of FM development. Second, the community is still investigating the proper pretraining tasks. Because transcriptomics data do not explicitly contain order information processed by architecture like transformer, the pretraining policy of developing an FM based on sequence data needs to be revisited. For example, the temporal and spatial trajectory [41] could be a new angle for designing a suitable pretraining task or architecture. Moreover, model development should fully consider the design of the benchmarking framework. A good FM should not only perform well in low‐level tasks but also inspire researchers to address high‐level tasks or even discover new biology. Finally, the budget for computation resources should also be taken into consideration. The cost of GPU hours as well as LLM API should be planned, and it will be helpful to open source a series of models with different scales [48] or an online‐access demo [49, 50, 51], which can enhance the accessibility of a true FM.

## AUTHOR CONTRIBUTIONS

**Tianyu Liu**: Conceptualization; investigation; resources; writing—original draft; writing—review and editing. **Minsheng Hao**: Resources; writing—original draft; writing—review and editing. **Xinhao Liu**: Resources; writing—original draft; writing—review and editing. **Hongyu Zhao**: Writing—review and editing.

## CONFLICT OF INTEREST STATEMENT

Hongyu Zhao is one of the Editorial Board Members of *Quantitative Biology*. He was excluded from the peer‐review process and all editorial decisions related to the acceptance and publication of this article. Peer review was handled independently by the other editors to minimize bias. The remaining authors declare no conflicts of interest.

## ETHICS STATEMENT

There is no ethnics issue.

## Data Availability

Data sharing is not applicable to this article as no new data were created or analyzed in this study.
